# Construction of nucleus-directed fluorescent reporter systems and its application to verification of heterokaryon formation in *Morchella importuna*

**DOI:** 10.3389/fmicb.2022.1051013

**Published:** 2022-11-21

**Authors:** Qianqian Zhang, Fang Shu, Xin Chen, Wei Liu, Yinbing Bian, Heng Kang

**Affiliations:** ^1^Institute of Applied Mycology, College of Plant Science and Technology, Huazhong Agricultural University, Wuhan, China; ^2^Hubei Hongshan Laboratory, Wuhan, China

**Keywords:** *Morchella importuna*, fluorescent protein, histone H2B, heterokaryon formation, *Agrobacterium*-mediated transformation

## Abstract

**Introduction:**

*Morchella importuna (M. importuna)* is a rare fungus with high nutrition value and distinct flavor. Despite the successful artificial cultivation, its genetic characteristics and biological processes such as life cycle, reproductive system, and trophic mode remain poorly understood.

**Methods:**

Considering this, we constructed pEH2B and pMH2B vectors by fusing *M. importuna* endogenous histone protein H2B with fluorescent proteins eGFP or mCherry, respectively. Based on the constructed pEH2B and pMH2B vectors, nuclear fluorescence localization was performed via *Agrobacterium tumefaciens*-mediated transformation (ATMT). These two vectors were both driven by two endogenous promoters *glyceraldehyde 3-phosphate dehydrogenase (GPD)* and *ubiquitin (UBI)*. The vector-based reporter systems were tested by the paired culture of two genetically modified strains pEH2B-labeled M04M24 (24e, MAT1-1-1) and pMH2B-abeled M04M26 (26m, MAT1-2-1).

**Results:**

The fluorescence observation and molecular identification results indicated the successful hyphal fusion and heterokaryon formation. We found that the expression of the reporter genes was stable, and it did not interfere with the growth of the fungus.

**Discussion:**

Our constructed nucleus-directed fluorescent systems in *M. importuna* can be used for monitoring the dynamic development and reproductive processes in living cells and also for monitoring the interaction between morels and plant roots. Therefore, morels exhibit the potential to be a candidate organism used for the research on basic biology and genetics of ascomycetes.

## Introduction

*Morchella importuna* has been successfully cultivated outdoors, and the rapid development of its cultivation process brings huge economic profits along with enormous risks due to the limited knowledge of its cognition of physiology, biology and genetics. Recently, the research on morels has mainly focused on taxonomy (Du et al., 2012; Baroni et al., 2018; Du et al., 2019; Machuca et al., 2021), cultivation technology (Liu Q. et al., 2018; Oys et al., 2021; Xu et al., 2022), multi-omics analysis (Liu W. et al., 2018; Murat et al., 2018; Han et al., 2019; Hao et al., 2019; Tan et al., 2019; Wang et al., 2019; Liu et al., 2020a,b; Cai et al., 2021), and microbial metabolism (Benucci et al., 2019; Longley et al., 2019; Tan et al., 2021a,b). However, the life cycle, trophic mode and reproductive mechanism of morels remain largely unknown due to the complicated species characteristics and difficulty in accomplishing the whole life cycle under the laboratory conditions. Fluorescent reporter systems are significant basis for the studies of fungal biology and genetics, and they have been broadly used in plants (Haseloff and Amos, 1995; Chiu et al., 1996), animals (Pines, 1995; Gubin et al., 1997), bacteria (Chalfie et al., 1994; Inouye and Tsuji, 1994) and fungi (Spellig et al., 1996; Rech et al., 2007; Kemppainen et al., 2020). So far, no valid fluorescent proteins (FP) reporter systems have been available in morels.

Fluorescent proteins (FP) are vital markers, and they can be used for investigating gene expression (Chalfie et al., 1994; Du et al., 1999), protein localization (Fernandez-Abalos et al., 1998), and fungal-plant interaction (Spellig et al., 1996; Vanden Wymelenberg et al., 1997; Maor et al., 1998; Kilaru et al., 2015). No exogenous substrate, or co-factors are required in fluorescence detection (Chalfie et al., 1994; Heim et al., 1994; Delagrave et al., 1995; Heim et al., 1995). In addition, the internal reporter requires no cell lysis or fixation, therefore allowing live-cell imaging. Two FP markers eGFP and mCherry were used in this study. eGFP (S65T) mutant derived from jellyfish *Aequorea victoria* (Cubitt et al., 1995; Chiu et al., 1996; Yang et al., 1996), whereas mCherry derived from *Discosoma* sp. (Shaner et al., 2004). Both were common reporter (Prasher et al., 1992; Chiu et al., 1996; Lorang et al., 2001) with high expression level, rapid chromophore formation, and relatively low photobleaching. However, the spontaneous background fluorescence often affects the observation effect, thus causing the inaccuracy of results. Histones are principal structural proteins on eukaryotic chromosomes. Recently, histones have been fused with fluorescent proteins (FP) to label nuclei in diverse filamentous fungi, with great signal stability throughout the life cycle (Maruyama et al., 2001; Freitag et al., 2004; Li et al., 2007; Rech et al., 2007; Kemppainen et al., 2020). The traditional cytoplasmic fluorescence is diffused in the whole cell, which is easily covered by the spontaneous fluorescence, and thus it is hard to be detected in the case of weak fluorescence expression. Compared with traditional cytoplasmic fluorescence, the nucleate-directed fluorescence exhibit great advantages in that the nuclear FP signal is more concentrated, so that it can be easily observed. In *Laccaria bicolor*, the introns of histone genes have been required for successful FP expression (Kemppainen et al., 2020). Additionally, effective FP expression is intron-dependent in some fungi (Burns et al., 2005; Ford et al., 2016). Therefore, we utilized the genomic gene sequences of *H2B*, rather than cDNAs, for FP fusion.

In this study, we fused *M. importuna* histone gene H2B (M04M26Gene04385) with eGFP (enhanced green fluorescence protein) or mCherry (red fluorescence protein) to construct nuclei-labelled vectors (pEH2B and pMH2B), since eGFP and mCherry both exhibited desirable fluorescence signals in *Laccaria bicolor* (Kemppainen et al., 2020). To drive our constructed target genes, we designed two *M. importuna* endogenous promoters *glyceraldehyde 3-phosphate dehydrogenase* (*GPD*) and *ubiquitin* (*UBI*) containing the first intron, since intronless transgenes may result in FP expression failure (Ma et al., 2001; Scholtmeijer et al., 2001; Godio et al., 2004; Kemppainen et al., 2020). In addition to efficient promoter, expression of FP genes also requires a transformation system. The genome sequencing of the *M. importuna* (Liu W. et al., 2018) and the existing transformation systems (Lv et al., 2018) make the fluorescence expression experiments on *M. importuna* possible. Due to its vegetative hyphal fast growth, and susceptibility to *Agrobacterium tumefaciens, M. importuna* is an ideal model organism for expressing FPs by *A. tumefaciens-*mediated transformation (ATMT) method.

*Morchella importuna* is considered to be heterothallic fungus (Chai et al., 2017; Du et al., 2017; Liu W. et al., 2018; Chai et al., 2019), and thus *M. importuna* individuals carrying the opposite mating-type genes are required to mate and complete the sexual reproduction (Metzenberg and Glass, 1990; Volk and Leonard, 1990; Coppin et al., 1997; Debuchy and Turgeon, 2006; Chai et al., 2022). Single-spore isolation was previously conducted in *M. importuna* to obtain ascospores (Liu W. et al., 2018), and ascospores harboring a single karyotype germinated rapidly under suitable conditions, which were designated as homokaryons (Volk and Leonard, 2018). This was in coincidence with one previous report that all the multinucleate ascospores are homokaryons by confocal scanning microscopy (He et al., 2017). On the contrary, another study has reported that both *mat1-1-1* and *mat1-2-1* genotype are detected from a small proportion of single-spore isolates, suggesting the existence of heterokaryotic ascospores in the monospore strain population (Liu et al., 2021). This evidence supports that there might exist a pseudohomothallism in *M. importuna* (Liu et al., 2021). Cytological studies have revealed that morels are capable of forming heterokaryon in life cycles (Volk and Leonard, 1990, 2018). In filamentous fungi, heterokaryon is formed by somatic cell fusion, thus establishing the interconnected mycelial network, which contributes to water and nutrition exchange, colonization and survival (Herzog et al., 2015). Cell nonself recognition is controlled by het loci in some species (Glass and Kuldau, 1992), but this is not the case for *M. importuna* (Chai et al., 2022) despite vegetative incompatible (VI) was ubiquitous in *M. importuna*. Besides, vegetative hyphae fusion (anastomosis) is common in *Morchella* (Volk and Leonard, 2018). After fusion, the two cell compartments can share nuclei and other organelles.

The mycelia of morels were separate, multicellular, multinuclear network, extended by tip growth, branching and fusion. Mycelia exhibit enormous difference in nucleus number with an average number of 10–15 nuclei per cell (Volk and Leonard, 1990). The functions of the multinucleate cells remain unclear, but become an obvious feature of *Morchella*, which is also common in other ascomycetes. Nucleus visualization of *M. importuna* was achieved by DAPI staining (He et al., 2017), and subsequently spatial and temporal distribution of nucleus has been examined by mating type gene analysis (He et al., 2021). It is worth noting that nucleus staining, such as DAPI and PI (propidium iodide) is toxic, and it affects the physiological activity of the target, and unavoidably causes environmental pollution. Therefore, nucleus staining is unsuitable to be used for living cells or organics. The morphological features of *Morchella* hyphae, spores, and even fruiting bodies vary greatly with the difference in the cultural medium components, temperature, time, and environment of culture (Hervey et al., 1978; Buscot, 1993; Kalyoncu et al., 2009). Such great difference is seldom observed in the macro-fungi during both vegetative stages and reproductive stages. Due to the multinucleate cells and changeable morphology, it’s difficult to distinguish the homokaryons from heterokaryons *via* morphological observation. Therefore, nucleus-directed fluorescence reporter system is essential to revealing the dynamic process of development and sexual reproduction of the living cells.

In present work, the systems of histone H2B–FP fusion expression in *M. importuna* was established. The fusion of histone and FP was initiated by GPD promoter from *M. importuna* and was terminated by TtrpC terminator from *Aspergillus nidulans*. The *hph* gene under the control of ubiquitin promoter from *M. importuna* to obtain hygromycin B resistance, and then it is used for transformant selection. We constructed two different nuclear fluorescence labeling vectors (pEH2B and pMH2B), and transformed them into *M. importuna* hypha using ATMT method to obtain transgene materials with visible nuclei. Genetically modified strains with different labels were paired-cultured in the petri dishes. Both green and red fluorescences of the hyphae were observed at the center of contact interface, indicting the hyphae fusion and heterokaryon formation. Our research provided nuclear-labeled mono-spore strains, allowing convenient and direct observation of nuclear behaviors, and they would be important materials for studying development and sexual reproductions of *M. importuna*. The targets of the study were: (1) to determine the expression of H2B fused FPs in *M. importuna* and to use them for monitoring the heterokaryon formation; and (2) to provide an applicable plasmid toolkit for fluorescent reporter system and to explore the potential of morels as candidate model species so as to reveal physiological and genetic characteristics of ascomycetes.

## Materials and methods

### Strains and culture conditions

Two single spore strains with opposite mating types were previously isolated from the cultivated strain M04 and designated as M04M24 (*MAT1-1-1*) and M04M26 *(MAT1-2-1)* (Liu W. et al., 2018). Both strains were generously provided by Liu W. et al. (2018) (Kunming Institute of Botany, Kunming, China), and stored in the Institute of Applied Mycology, Huazhong Agricultural University (Wuhan, China). The strains were cultured on the complete yeast extract medium (CYM, containing 20 g Glucose, 2 g Peptone, 2 g Yeast extract, 0.5 g MgSO4·7H_2_O, 1.0 g K_2_HPO_4_, 0.46 g KH_2_PO_4_, 2% Agar) in 9-cm petri dish at 23°C in darkness. After 5-day culture, the mycelium blocks were removed into new CYM medium plate covered with sterile cellophane membrane and cultured for 7 days at 23°C. The hyphae were collected, quickly frozen by liquid nitrogen, and stored at the −80°C for subsequent genomic DNA extraction (Doyle, 1987). Genetically modified morel strains were cultured on CYM medium added with 10 μg/ml of hygromycin B under the same conditions. *Escherichia coli* strain Trans1-TI (TransGen Biotech, Beijing) serving as the host for general plasmid construction was cultured on Luria–Bertani (LB) medium supplemented with 50 μg/ml kanamycin at 37°C overnight. The *Agrobacterium tumefaciens* strain EHA105 was used for transformation of *M. importuna*, and grown on LB medium added with 50 μg/ml kanamycin (kana) and 20 μg/ml rifampicin (rif) at 28°C for 2–3 days. The plasmids were transformed into *E. coil* and *A. tumefaciens* according to the manufacturers’ instruction.

### Construction of pEH2B and pMH2B vector

The plasmid was constructed by homologous recombination methods (Sambrook et al., 1989). The reagents were purchased from TransGen Biotech (Beijing, China) and Thermo scientific (Shanghai, China). The primers used in this study were designated by CE design V1.02 and the sequence were listed in Table 1. Primers were synthesised by Tsingke Biotechnology (Beijing, China). After the homologous recombination, the recombinant plasmids were transformed into *E. coli* strain Trans1-TI for plasmid replication. In order to express H2B-eGFP and H2B-mCherry fusion protein for nucleus visualization in *M. importuna* strains, the entire coding sequence of histone H2B (MIM04M26Gene04385) were amplified from gDNA of M04M26. H2B fragment with introns but without stop codon was fused with eGFP or mCherry at C-terminus for nuclear localization (Kemppainen et al., 2020).

**Table 1:** Primers used in this study.

Primer name	Primer sequence (5′-3′)
eH2B-F	cactaaatagtcaaagGATCCCCTTCGTCGATAATCCAAAACC
eH2B-R	gtccttgtagtccatcccGGGTTTGGTGGAAGAAGAGTACTTGGTG
mH2B-F	caaaCCTTCGTCGATAATCCAAAACC
mH2B-R	gctcaccatacctgcaatcccGGGTTTGGTGGAAGAAGAGTACTTGGT
Ubiquitin-F	gcaatcgatggcgataAGCTTGATGGTGGGTATTGAGTTTAAGAATG
Ubiquitin-R	ctggggtgcctaatgTATGTAGAATAAGAAGTGCTTCATAGATGC
Link-F	ctacataCATTAGGCACCCCAGGCTT
Link-R	ctccccgtacctcccAATATTCTCCCCGTACCTCCCCT
Gpd-F	ggagaatattGGGAGGTACGGGGAGAATATTTTG
Gpd-R	ggattatcgacgaaggTTTGACTATTTAGTGAATCTATTTAACGCA
eyz-F	TAGGAAGCCGGAAAGAAGACT
eyz-R	CGCTTCTCGTTGGGGTCTTT
myz-F	TCCGTCAGGACATTGTTGGAG
myz-R	TACCATTTTCGGCCTATCAGA
hpt557-F	ACACTACATGGCGTGATTTCAT
hpt557-R	TCCACTATCGGCGAGTACTTCT
Mat1-1-1F	AAAACGCTCTATCGCCACCA
Mat1-1-1R	TGCGAAGTTGCGTTTTCAGG
Mat1-2-1F	ATGAAGGCTGTTCGCCAGAA
Mat1-2-1R	TGGTGCTCTCGTGCAGATTT

The sequences with lowercase characters indicate 5′ overhang of primers used for vector construction.

**Figure 1:**
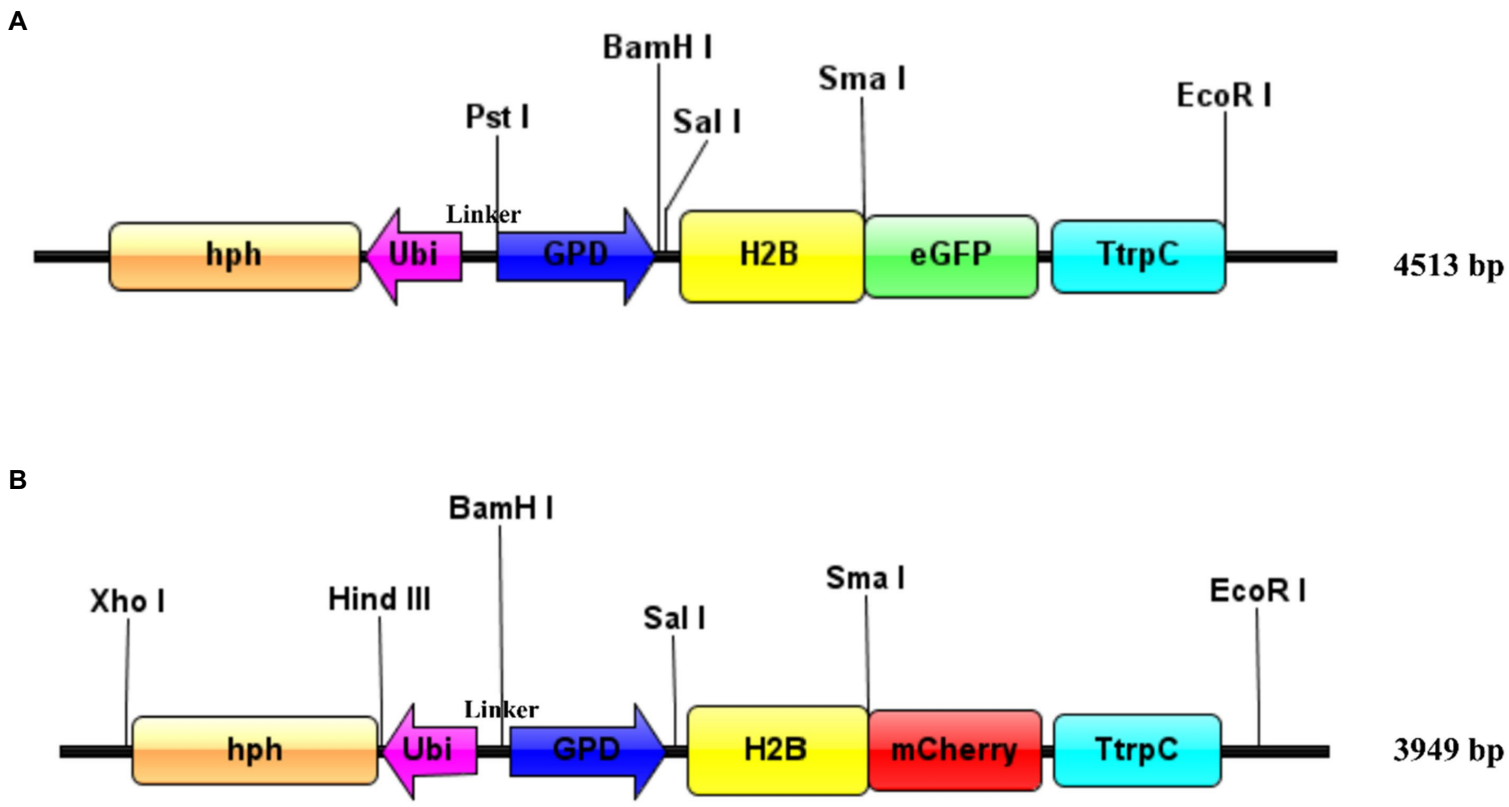
Schematic of plasmid design for transformation. **(A)** pEH2B vector. Enhanced green fluorescent protein was used as reporter gene. **(B)** pMH2B vector. MCherry was used as reporter gene. Both the vectors carrying *Morchella importuna* histone H2B (M04M26Gene04385) genomic sequence were fused with eGFP or mCherry. The fused fluorescent gene expression was initiated by the GPD promoter from *M. importuna* and terminated by the tryptophan synthase gene terminator from *Aspergillus nidulans.* The hygromycin resistance gene (hph) expression was initiated by the *M. importuna* ubiquitin (ubi) promoter. Unique restriction enzyme recognition sites used for linearization were marked above the vectors, and the vectors are easy to modified in the restriction site. The schematic was generated by IBS web server (http://ibs.biocuckoo.org/online.php; Liu et al., 2015).

The pEH2B vector (Figure 1A) was constructed based on the *M. importuna* binary vector p1391-U-GUS (Lv et al., 2018). The vector p1391-U-GUS was linearized by restriction enzymes *Sma* I (Thermo scientific). Then, the H2B fragment (745 bp) was amplified with primers eH2B-F/eH2B-R from gDNA of *M. importuna*, and inserted into the N-terminus of eGFP. Similarly, the pMH2B vector (Figure 1B) was constructed based on hyg-mCherry vector, which was obtained by replacing eGFP fragment of p1391-U-GUS vector with mCherry (Lv et al., 2018). For the construction of pMH2B vectors, the hyg-mCherry vector was cut by *Sma* I and *Hind* III (Thermo scientific). The H2B fragment (745 bp) was amplified with primers mH2B-F/mH2B-R from the gDNA of *M. importuna,* and inserted into the N-terminal of mCherry. The glyceraldehyde-3-phosphate dehydrogenase (*GPD*) promoter (655 bp upstream of the start codon) of *M. importuna* strain M04M26 was used to initiate H2B-eGFP/mCherry genes encoding the green/red fluorescent protein for labelling nuclei. The *ubiquitin (Ubi)* promoter (396 bp) of *M. importuna* was amplified with primers Ubiquitin-F/Ubiquitin-R, and was used to initiate the hygromycin B resistance cassette in opposite direction for the selection of transformants. The primer pair Link-F/R was used to amplify the linker sequence between *Ubi* promoter and *GPD* promoter. The schematic of constructed vectors (Figures 1A,B) was generated by IBS web server^1^ (Liu et al., 2015).

### *Agrobacterium*-mediated transformation of *Morchella importuna*

Transformation of *M. importuna* strains M04M24 and M04M26 was carried out by ATMT methods with minor modifications (Lv et al., 2018). The modifications were as follows: (1) We used receptor mycelia cultured for 2 days under 23°C, rather than for 5 days under 22°C because the younger mycelia had thinner cell wall, and thus more convenient for transformation (Sullivan et al., 2002; Michielse et al., 2005). (2) We skipped the homogenization steps including grinding the germinated mycelium blocks into fragments and homogenizing them for further selection. Grinding and homogenization might result in mycelium lesion, thus reducing the germination rate (Lv et al., 2018). In addition, the homogenization process might also cause the homogeneity of transformants derived from the same transformation events, thus lacking diversity. Here, we used a sterile puncher to punch germinated mycelium blocks and transferred them to a new medium for screening. After the third rounds of antibiotic screening, the concentration of hygromycin increased from 10 to 12 μg/ml to obtain more purified transformants. After 4 weeks of resistance screening, the colonies geminate in the hygromycin medium were considered as candidate positive transformants, which were stored at 4°C for subsequent experiment. Genomic DNA was extracted from candidate transformants using CTAB lysis buffer, and proteins were eliminated using phenol solution (PCI, phenol-chloroform-isoamyl alcohol mixture). Afterwards, nucleic acids were precipitated with 2 volumes of ethanol. RNA was removed after 1% RNAase A digestion for 1 h at 37°C to obtain pured DNA. Diluted DNA samples were stored at −20°C for further analysis.

### PCR identification and Southern blot assay

The primer pair eyz-F/eyz-R was designed to amplify H2B-eGFP fragment used gDNA of strain M04M24 transformants as the template. Similarly, primer pair myz-F/myz-R was used to amplify the H2B-mCherry fragment with the gDNA of strain M04M26 used as template. The primer sequences were listed in Table 1. PCR amplification was carried out with 10 μl of 2 × Taq Master Mix (Dye Plus) (Vazyme, Nanjing, China), 50 ng/ml genomic DNA, 1 μl of each primer (10 μΜ), and add ddH2O to a total volume of 20 μl. The PCR was performed as follows: 95°C for 5 min, followed by 30 cycles of 95°C for 30 s, 55°C for 30 s, and 72°C for 75 s. After the reaction, the products were examined with a 1% agarose gel. In order to determine whether the T-DNA fragment was inserted into the chromosome of *M. importuna*, Southern blot assay of four transformants selected by PCR was conducted. The mycelia of *M. importuna* were collected and high purity DNA was extracted using the Fungal Genomic DNA Rapid Extraction Kit (Sangon Biotech). *Hph* fragment was amplified with primer pair hpt557-F/R as a probe, and the gDNA of all the samples were digested with Hind III at 37°C for 1 h. The Southern blot assay was conducted with DIG High Prime DNA Labeling and Detection Starter Kit I (Roche, Germany) according to the manufacturer’s instructions.

### Fluorescence microscopy observation and mating type detection of heterokaryotic strains

The images of transformants were observed with a confocal microscope (Leica TCS SP8, Germany) and processed with LAS AF lite platform. Specifically, green fluorescence was observed at 488 nm excitation wavelength, and red fluorescence was observed at 543 nm excitation wavelength. Untransformed strains were used as control. Meanwhile, mycelia were stained using 5 μg/ml DAPI solution (Sigma-aldrich, United States) for nucleus observation. Two different fluorescently labeled-homokaryon strains were paired cultured in CYM medium at 23°C in darkness for 4 days, and then two 5 mm mycelium blocks were put at the opposite edge of a petri dish covered with CYM medium for culture. The mycelium blocks in the contacted area were cut into pieces, and transferred into petri dish supplement with CYM medium. After germinating, the tip of mycelium was picked to obtain pure culture. Then, the mycelia were collected for DNA extraction. PCR amplification was conducted with mating type gene primers. The strains containing both mating type locus were considered as hybrid strains. The mycelia of hybrid strains were transferred into a new petri dish to obtain pure culture. The gDNA was extracted from the hybrid strains, as described in see section “PCR identification and Southern blot assay.” Mating type primers Mat1-1-1F/R and Mat1-2-1F/R (Table 1) were used to detect the mating type genes, and the fluorescence was observed as mentioned above.

## Results

### GPD promoter drive H2B-FP fusion expression

**Figure 2:**
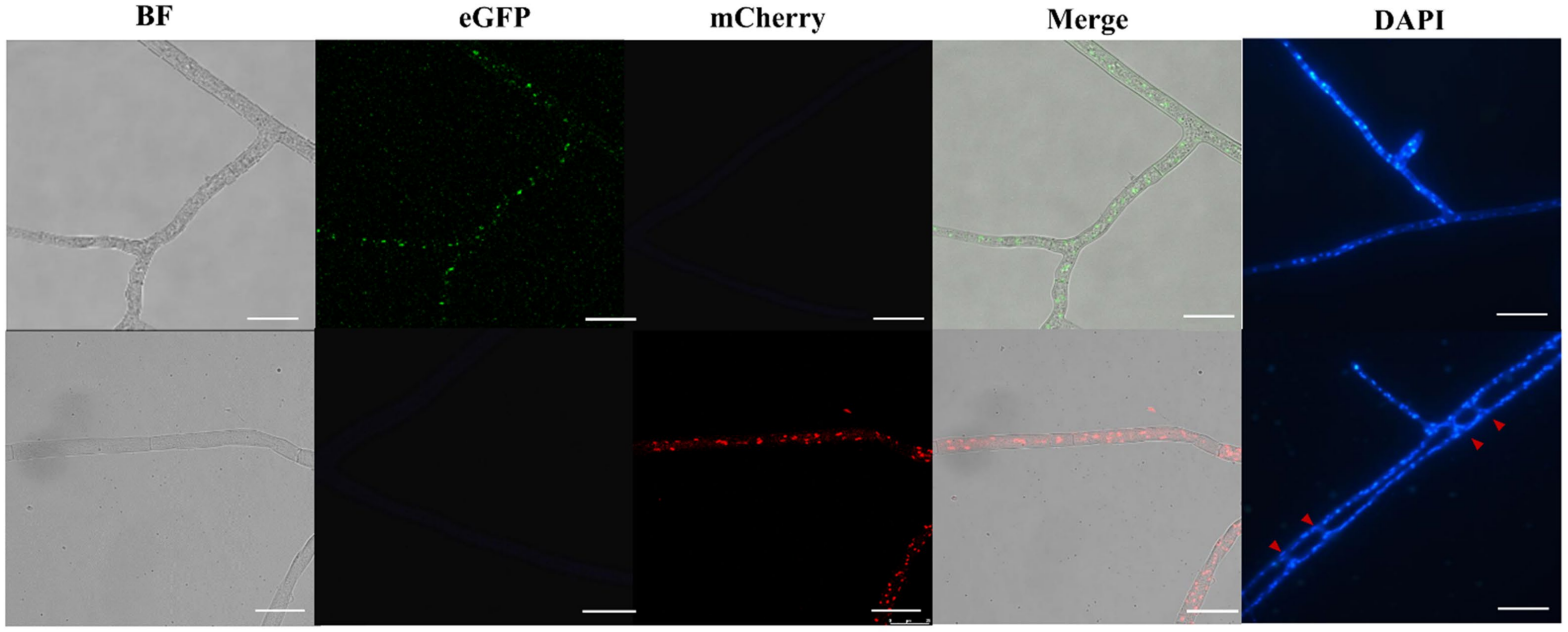
Fluorescence microscopic observation of *M. importuna* transformants. Green (H2B-eGFP) or red (H2B-mCherry) dots indicate the location of nuclei. DAPI-stained nuclei supported the corrected expression of H2B-FP, and red arrows point to vegetative fusion of two adjacent hyphae. BF, bright field; Merge, tricolor overlapping of BF, eGFP, and mCherry. Bar = 25 μm.

To obtain stable and visible nuclei fluorescence, histone H2B gene of *Morchella importuna* was used to construct nuclei fluorescent-labeled vectors. We constructed two vectors pEH2B (Figure 1A) and pMH2B (Figure 1B) encoded H2B fused with eGFP or mCherry in C-terminal, and exhibited green and red nuclear fluorescence, respectively. We utilized the genomic sequences of H2B (745 bp), rather than cDNAs, for fluorescence protein fusion. GPD promoter has been used widely to express FP genes (Murata et al., 2006; Ding et al., 2011), therefore, we employed the GPD promoter from *M. importuna* to drive the expression of fusion genes H2B-eGFP and H2B-mCherry. Vector pEH2B and pMH2B were, respectively, transformed into M04M24 (Mat1-1-1) and M04M26 (Mat1-2-1) strains of *M. importuna* by ATMT method (Supplementary Figure 1). After 4–5 rounds of screening, we observed the normal growth of the strains grown on the resistant plate, thus these strains were determined as positive transformants. As we expected, we also observed green and red nuclear fluorescence signals at the 488 nm in nuclei of strain M04M24 and 543 nm in nuclei of strain M04M26 under the Leica confocal microscope, respectively, indicating the fusion proteins H2B-eGFP and H2B-mCherry were successfully expressed (Figure 2). However, only weak autofluorescence was detected from cytoplasm of the wildtype strain with no dotted nuclei signal visible. Furthermore, our DAPI staining results showed successful nuclear FP localization in the target strains (Figure 2). In this study, we obtained H2B-eGFP labelled M04M24 strains, and H2B-mCherry labelled M04M26 strains, both showed stable nuclei fluorescence signal. Overall, two target strains with different mating type genes were successfully labeled by FP in hyphal nuclei, indicating FP genes were functionally expressed in *M. importuna*.

### Transgene fragments were identified by PCR and Southern blot

**Figure 3:**
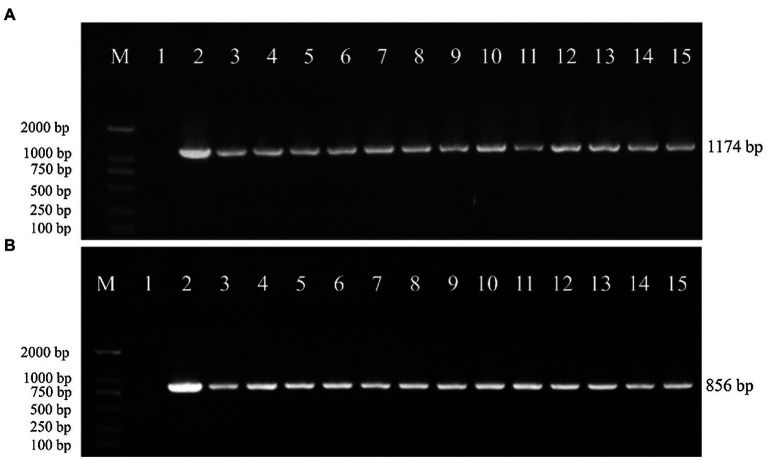
Genomic DNA extraction and PCR identification of *M. importuna* transformants. **(A)** M04M24 strain transformant transformed with pEH2B vector. Primers eyz-F/R were used to amplify H2B-eGFP insertion fragment. Lane1, untransformed M04M24 strain; Lane2, pEH2B plasmid; Lane3-15, randomly selected transformants of pEH2B. **(B)** M04M26 strain transformant transformed with pMH2B vector. Primers myz-F/R were used to amplify H2B-mCherry insertion fragment. Lane1, untransformed M04M26 strain; Lane2. pMH2B plasmid; Lane3-15, randomly selected transformants of pMH2B. M, BM2000 marker (Biomed, Beijing, China). The expected size of PCR products is indicated on the right.

**Figure 4:**
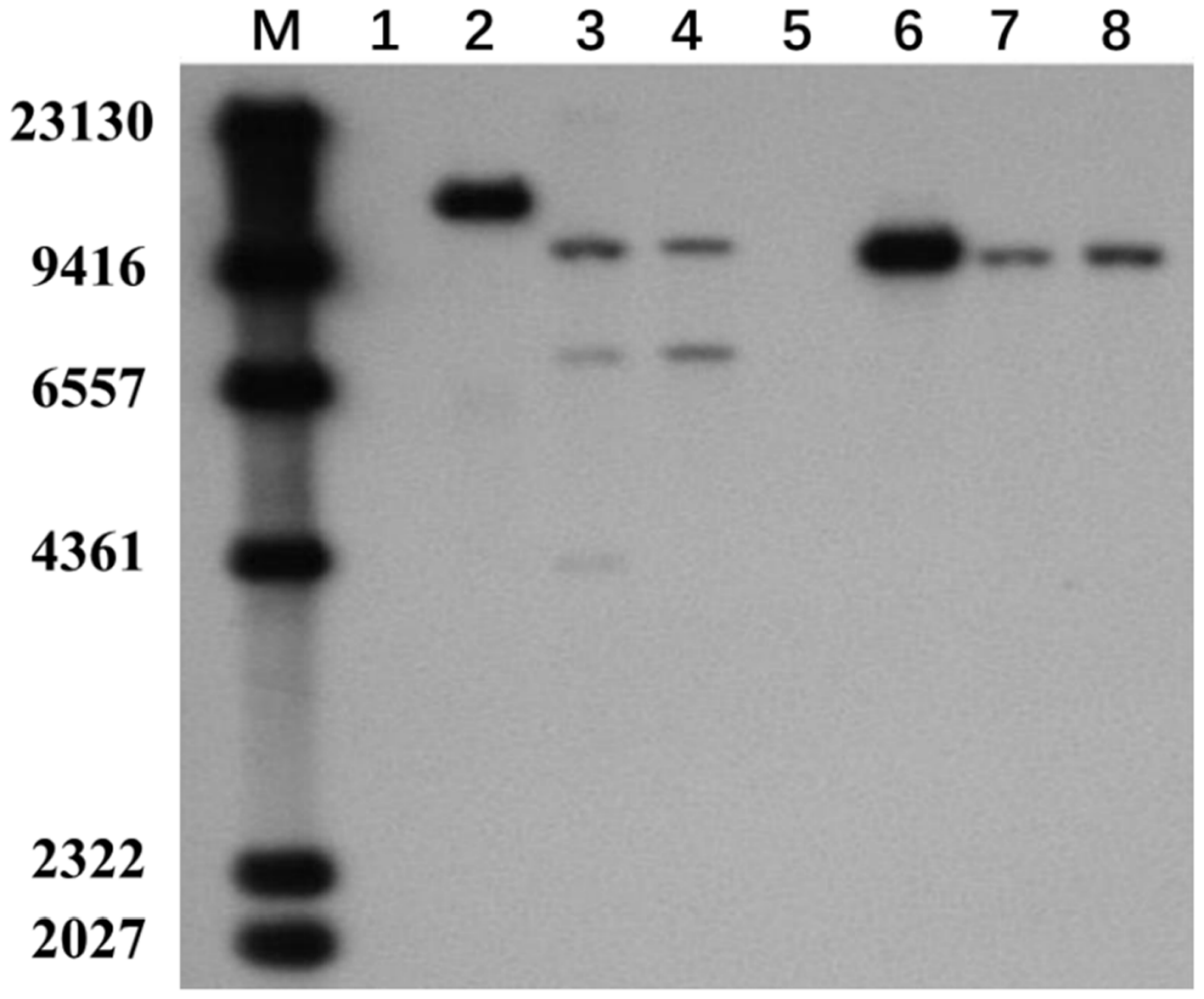
Southern blot assay of *M. importuna* transformants. M, maker. Lane1, untransformed M04M24 strain as negative control; Lane2, pEH2B plasmid as positive control; Lanes3, 4, randomly selected transformants of pEH2B; Lane5, untransformed M04M26 strain as negative control; Lane6, pMH2B plasmid as positive control; Lanes7, 8, randomly selected transformants of pMH2B. The numbers on the left indicate the base pair (bp) number of markers.

To verify whether the transgenic strains transformed with the pEH2B and pMH2B vectors were correctly integrated into the genomes of target strains, we performed PCR amplification and Southern blot analysis of candidate genes. We obtained a large number of hygromycin-resistant candidate transformants for PCR identification, from which we arbitrarily selected 13 positive transformants to display the results of PCR amplification (Figure 3). Wildtype strains M04M24 and M04M26 were used as negative control. The PCR results showed that the size of amplified band (1,174 and 856 bp) was consistent with the expected fragment sizes (Figures 3A,B), suggesting the plasmids were successfully integrated into the genome of *M. importuna*. Subsequently, four transformants were selected by Southern blot analysis with *hph* gene as the probe. Untransformed strains M04M24 and M04M26 were used as the negative control, and fused plasmids pEH2B and pMH2B were used as the positive controls (Figure 4). The Southern blot results indicated that target gene insertion was detected from all the four transformants, but not detected from untransformed strains (wildtype) M04M24 and M04M26 (Figure 4). Besides, two transformants of fused plasmid pMH2B presented one single copy at the same insertion site, while transformants of fused plasmid pEH2B exhibited 2 or 3 inserting sites with hph fragment as the probe.

### Vegetative compatible strains form heterokaryon

**Figure 5:**
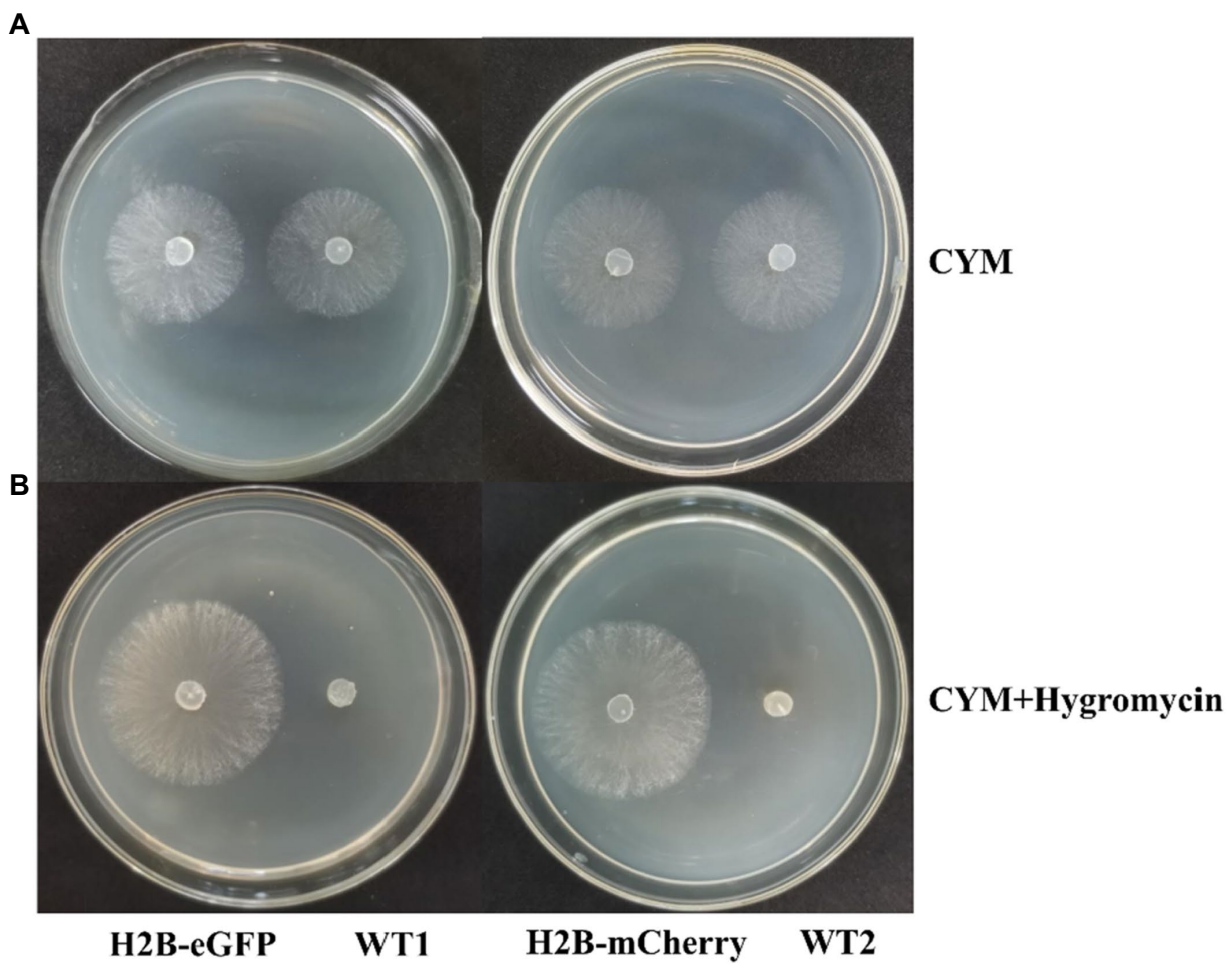
Expression of H2B-EGFP/mCherry does not influence hyphae growth. **(A)** Growth of H2B-EGFP/mCherry strains and wild type strains in CYM medium for 2 days at 23°C. **(B)** Growth of H2B-EGFP/mCherry strains and wild type strains in CYM medium supplemented with 10 μg/ml hygromycin B for 3 days at 23°C. Two WT strains showed no growth under *hph* gene screening. H2B-EGFP, M04M24 transformant containing pEH2B plasmid; WT1, untransformed M04M24; H2B-mCherry, M04M26 transformant containing pMH2B plasmid; WT2, untransformed M04M26.

To examine the stability of the FP signals and the effect of fused genes on fungal growth, we tested the growth of the transgenic strains and wildtype (WT) strains in CYM medium. We found no significant difference between them (Figure 5A). After 5 days of growth, mycelia covered all over the 9 cm petri dish, and aerial hyphae began to appear, which was consistent with WT strains. In CYM medium supplemented with 10 μg/ml of hygromycin B, neither WT strains (M04M24 and M04M26) exhibited growth, whereas transformants showed normal growth (Figure 5B). To examine the stability of transformants, the hypha blocks of wildtype and transgenic strains were stored for 4 months at −80°C in the 50% sterile glycerol-water solution (1:1, v/v). The results showed that mycelia could still geminate and recover to normal growth state on the CYM medium (Supplementary Figure 2). After 3-day culture, screening was performed by *hph* resistance. We found that the nucleus fluorescence signal was still intense under the confocal microscope, indicating that fused protein transformation had no negative influence on the hyphae growth, and the FP signal was stable.

**Figure 6:**
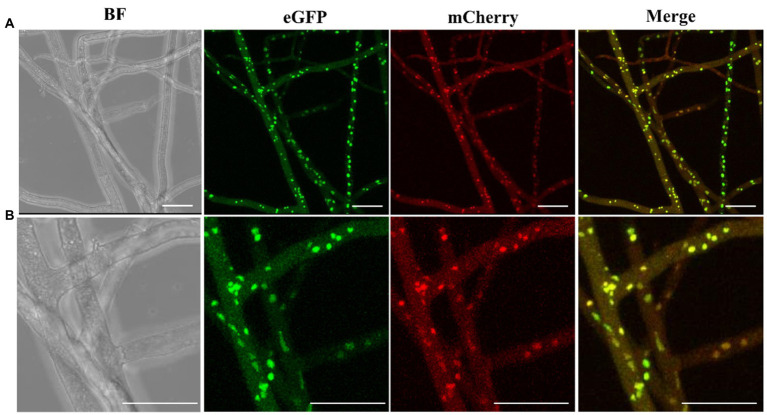
Fluorescence microscopic observation of nucleus confirms heterokaryon formation. **(A)** eGFP or mCherry fluorescence signals of *M. importuna* hyphae at day 5 post culture in the CYM medium supplemented with 10 μg/ml hygromycin. eGFP exhibited a stronger signal intensity than mCherry. The overlapping of red and green signals occupied most area. **(B)** The enlarged images of **(A)**, the yellow fluorescence signal represents the overlapping of red and green signals. BF, bright field. Scale bars = 25 μm.

**Figure 7:**
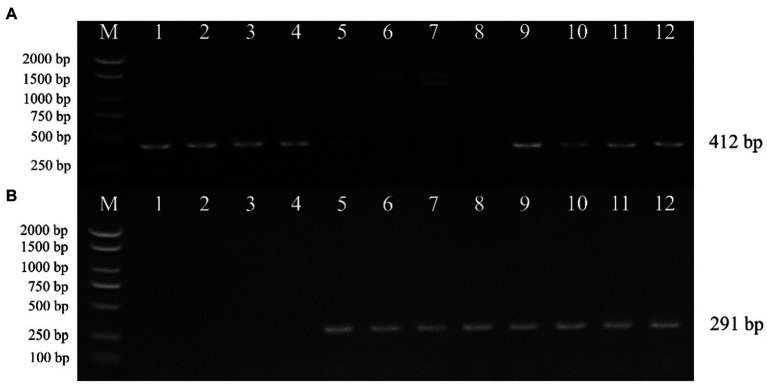
Mating type genes amplification of *M. importuna* transformants by PCR. Candidate heterokaryon strains were formed by paired culture of transformants harboring opposite mating type genes. **(A)** *MAT1-1-1* gene amplification. **(B)** *MAT1-2-1* gene amplification. Lanes1–4, M04M24 strains with the insertion of pEH2B plasmid, showed mat 1-1-1 genotype; Lanes 5–8, M04M26 strains with the insertion of pMH2B plasmid, showed mat 1-2-1 genotype; Lanes 9–12, randomly selected candidate heterokaryon strains, both mating type genes were detected. M, BM2000 marker (Biomed, Beijing, China). The expected size is indicated on the right.

To investigate the heterokaryon formation, we performed paired culture of transgenic strains M04M24 and M04M26. As a result, we observed both green and red fluorescence (Figures 6A,B) in the nuclei of hyphae. The bicolor (green and red) merged images showed a large proportion of bicolor fluorescence overlap, which took on yellow (Figures 6A,B) The enlarged images (Figure 6B) displayed that two fluorescence signals were clearly merged with each other, suggesting that cell fusion occurred between two vegetatively compatible strains, and these fused cells shared the same cytoplasm and organelle. Both the fluorescence proteins (eGFP and mCherry) were expressed in cytoplasm and localized into the same nucleus, indicating the formation of heterokaryons. Besides, eGFP exhibited a higher fluorescence intensity than mCherry in *M. importuna* mycelia (Figures 6A,B). The heterokaryons were further identified by the mating type amplification. PCR amplification results showed that heterokaryon included both mating types (Figure 7), which was in coincidence with the fluorescence observation results under microscope (Figures 6A,B).

## Discussion

*Morchella importuna* has been reported to be heterothallic fungus (Chai et al., 2017; Du et al., 2017; Liu W. et al., 2018; Chai et al., 2019), and two opposite mating types are required to complete its sexual reproduction (Metzenberg and Glass, 1990; Coppin et al., 1997; Debuchy and Turgeon, 2006; Chai et al., 2022). However, lack of basic biology information on *M. importuna* caused a serials of unsolved problems, such as the unstable yields in outdoor cultivation, strain degeneration, trophic mode controversy and ambiguous life cycle, which poses the obstacles for the development and research on morels in spite of mature cultivation technology. Fluorescence reporter system is a vital tool with an extensive range of application. The increasing published omics data of some morel species make functional genomic analysis possible (Liu W. et al., 2018; Murat et al., 2018; Han et al., 2019; Hao et al., 2019; Tan et al., 2019; Wang et al., 2019; Liu et al., 2020a,b). Up to now, various tools and strategies for filamentous fungus function analysis have been developed, since the first DNA transformation experiment was reported in *Neurospora crassa* (Mishra and Tatum, 1973). Undoubtedly, these works will contribute to understanding and utilization of filamentous fungi, since they are desirable model materials due to their simple cellular structure and small genome size. The genetic transformation system is the foundation of gene function study. Compared with the research on model filamentous fungi (van de Rhee et al., 1996; de Groot et al., 1998; Mikosch et al., 2001; Michielse et al., 2004), the gene functional analysis of morel started relatively late, with the first *Agrobacterium tumefaciens*-mediated transformation method in 2018 (Lv et al., 2018). In the ATMT method, GFP was firstly used as a reporter gene to screen transformants, as a result, 18 out of 22 positive transformants were detected to have fluorescence in mycelia cytoplasm, and the remaining 4 positive transformants lose their fluorescence signal, which might be due to the presence of chimeras (Lv et al., 2018).

In this study, we firstly cloned the *H2B* gene of *M. importuna,* fused it, respectively, with eGFP or mCherry to label the nuclei of two single spore strains by ATMT method, and further verified the stability. Compared with the cytoplasm fluorescence (Lv et al., 2018), nuclei fluorescence was more focal, more stable, and less affected by autofluorescence, and thus it can be applied in more situations. He et al. (2021) investigated the nuclear distribution of *M. importuna* by mating gene amplification, found that nuclear was unevenly distributed in the cells, and that Mat1-1-1 was the dominant genotype (He et al., 2021). This was consistent with the report by Du et al. (2017), who found that Mat1-1-1 showed competitive advantage in natural populations (Du et al., 2017). The requirement for both two opposite mating types involves enormous extra work, since the common uneven distribution of mating types in morels. In addition, it is hard to distinguish mating types *via* morphology observation of primary and secondary mycelium in ascomycetes like morels. These factors pose giant barriers for morels breeding. In order to facilitate the morel breeding development, we established two efficient fluorescent vectors with red and green fluorescence to label nuclei, and further used them for quickly and accurately screening stable heterokaryon. The fluorescence of H2B-eGFP and H2B-mCherry were present in nuclei throughout the cell cycle, allowing the observation of the dynamic nucleus division in living cells. Meanwhile, we employed endogenous GPD promoter of *M. importuna* containing the first intron to drive FP expression and found that this GPD promoter of *M. importuna* produced sufficient transformants. Our finding was in line with the study results of other species (van de Rhee et al., 1996; Shi et al., 2012; Zhang et al., 2014).

Currently, few studies have investigated the application of nucleus-directed fluorescence reporter system in morel, which might be attributed to the low efficiency of transformation and the presence of high proportion of chimeras (Lv et al., 2018). Considering this, we modified the existing transformation systems. Firstly, we selected young hyphae as the recipient since the cell wall of filamentous fungi is the main obstacle for a successful genetic transformation. In existing studies, protoplasts are usually used as receptor cells of filamentous fungi, but low protoplast regeneration in *Morchella* tends to reduce the transformation efficiency (He et al., 2020). Secondly, we canceled the grinding steps because manual grinding might do damage to mycelia, thus lowering germination rate. Further, we screened more mutant individuals at different insertion sites from extensive positive transformation events to ensure the diversity of transformants. Thirdly, in *Agrobacterium tumefaciens*-mediated transformation, different *Agrobacterium tumefaciens* strains AGL-1 (Lazo et al., 1991) and EHA105 (Wirawan et al., 1993), harboring the plasmid pEH2B and pMH2B were employed. The result indicated that AGL-1 was not suitable for *M. importuna* transformation system with a transformation efficiency of 0%, while EHA105 performed much better with a transformation efficiency of 40% (data not shown), which might be ascribed to the difference of infection ability of *A. tumefaciens* strains. Our results were in line with the previous reports on *Hypsizygus marmoreus*, that no positive transformants were obtained from AGL-1- mediated transformation when protoplast was used as the receptor (Zhang et al., 2014). However, AGL-1 has been reported to be widely used in some species, and it even performs better than EHA105 (Zhang et al., 2011), and conidia were used as the receptor cells in their studies (Jeon et al., 2007; Ji et al., 2010; Zheng et al., 2011), which might explain the different findings. Although conidia are desirable materials for transformation, however, it is hard to collect condia from *M. importuna* under controlled conditions. Since the concerning mechanism remains unknown, we suggested screening appropriate Agrobacterium strains and receptor cells before the transformation experiment.

Hyphal fusion (anastomosis) is an important event in the fungal life cycles, which has been well studied (Rech et al., 2007; Fleissner and Herzog, 2016). Hyphal fusion of *Morchella* has been studied cytologically, and the micrographs reveal heterokaryon formation and high cell fusion frequency (Hervey et al., 1978; Volk and Leonard, 2018). The formation of heterokaryons result in functional diploidy and genetic complementation, which is crucial for colony development and genetic diversity, especially for those of complicated eukaryote. Liu et al. (2021) used homokaryonic strains with opposite mating types to conduct confrontation culture and found that vegetative incompatibility was ubiquitous in *Morchella* (Liu et al., 2021). Currently, there are few reports on cell fusion and heterokaryon formation of *M. importuna*. Considering this, we assessed the heterokaryon formation by paired culture of two opposite mating type strains labeled with different nucleus-directed fluorescence reporter genes. Our data showed that the compatible homokaryotic strains mated by hyphal fusion, and two colors coexist in the same nuclei of mated hyphae. Although, we are unable to distinguish the ploidy level of the mated strain based on the existing data, it is impossible for morels to form diploid nuclei in vegetative growth stages according to the existing research (Du et al., 2017; Du and Yang, 2021). Du et al. summarized the life cycle of morels, which is dominated by a haploid life cycle (Du and Yang, 2021). Diploid nucleus formed by karyogamy only occurs in the sexual process with a short lifetime, because meiosis takes place almost immediately after karyogamy to form haploid nuclei (Du et al., 2017; Du and Yang, 2021). Considering this, the possible explanation for the colocalization of two fluorescent proteins in the same nucleus is that the fluorescence proteins (eGFP and mCherry) were both expressed in cytoplasm and localized into the same nucleus by nuclear localization signal. The stable heterokaryotic strains were purified through tip hyphae culture, since tip hyphae only contain 1–2 nuclei (Volk and Leonard, 1990, 2018). In addition, the fluorescent signal of heterokaryotic strains was stable in hyphae according to our results.

Multiple factors can influence the efficiency of the genetic transforamtion, such as vectors, promoters, marker genes, screening system and receptor strains. The observation of nucleus dynamics is essential to the study of fungal cell biology, and nucleus fluorescent labeling does not interfere with the normal physiological state of the cell, and thus it allows continuous observation of the nucleus dynamics. However, transformation parameters such as co-cultivation time, PH, and concentration of acetosyringone needs systematic optimization in the future study. In addition, the extensive applicability of the fluorescent reporter system remains to be further verified. All in all, the construction of the *M. importuna* nuclear-direct fluorescent reporter system allows dynamically monitoring nuclear behaviors of living cells and fungi-plant interaction. For example, the FP-labeled mycelia can be co-cultured with plant roots to monitor the dynamic interaction process, which might contribute to understanding complicated trophic issues. Considering the rapid growth and easy culture of their vegetative hypha, morels can be used as a model organism for the studies of symbiosis mechanism.

## Conclusion

We firstly made the first attempt to cloned the histone H2B gene of *M. importuna* to co-expressed it with eGFP or mCherry, and obtained two fused vectors pEH2B pMH2B, based on which, we constructed stable nucleus-directed fluorescence reporter system by optimized ATMT method. We detected heterokaryon formation by paired culturing genetically modified strains harboring opposite mating type genes. The results indicated that vegetative hypha fusion was common in vegetative growth stages, and sexual compatible combinations in this experiment exhibited vegetative compatibility. Our findings of fluorescent protein expression in *M. importuna* contribute to the studies of hypha behavior and nucleus behavior in living organisms, molecular genetics, and morel-host root interaction. Our constructed FP reporter system provides an insight into morel genetics, physiology, and biology of morels.

## Data availability statement

The original contributions presented in the study are included in the article/Supplementary material, further inquiries can be directed to the corresponding author.

## Author contributions

QZ analyzed the data, and wrote and edited the revised manuscript. FS conducted the experiments and wrote the original manuscript. XC participated in fluorescence observation. WL generously provided strains and analyzed sequence of H2B. YB designed the experiments. HK supervised the experiments, provided guidance of the whole experiment, and funding support. All authors contributed to the article and approved the submitted version.

## Funding

This research was funded by the National Natural Science Foundation of China (grant no. 32070095) and HZAU-AGIS Cooperation Fund (JCKY2020-60 and 2662022JC003).

## Conflict of interest

The authors declare that the research was conducted in the absence of any commercial or financial relationships that could be construed as a potential conflict of interest.

## Publisher’s note

All claims expressed in this article are solely those of the authors and do not necessarily represent those of their affiliated organizations, or those of the publisher, the editors and the reviewers. Any product that may be evaluated in this article, or claim that may be made by its manufacturer, is not guaranteed or endorsed by the publisher.

## Supplementary material

The Supplementary material for this article can be found online at: https://www.frontiersin.org/articles/10.3389/fmicb.2022.1051013/full#supplementary-material

## Footnotes

^1^ http://ibs.biocuckoo.org/online.php
