# Supplementary material for: Construction of nucleus-directed fluorescent reporter systems and its application to verification of heterokaryon formation in Morchella importuna
Source: Front Microbiol. 2022 Nov 21;13:1051013. doi: 10.3389/fmicb.2022.1051013 (PMC9720127; doi:10.3389/fmicb.2022.1051013)
Supplement: Supplementary file 1 [file Data_Sheet_1.PDF]

Supplementary Material

1 Supplementary Data

1.1 Supplementary Figures

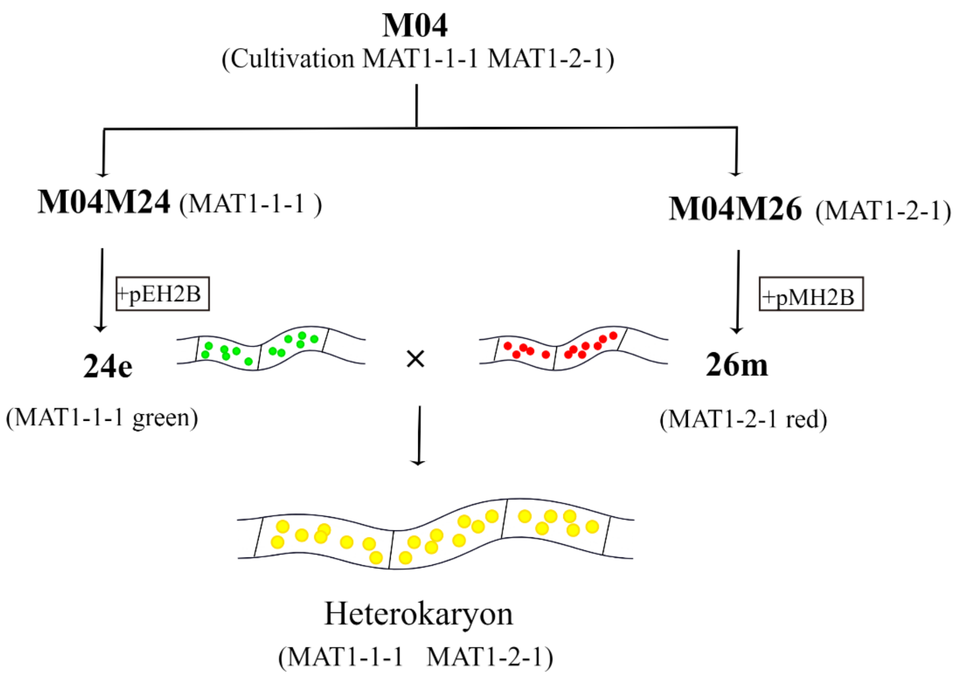

Supplementary Figure 1. Supplementary Figure 1. Schematic of strains used in this study. Single spore NO.24 and NO.26 was isolated from the cultivated strains M04 belonging to *Morchella importuna*, designated strain M04M24 (mat1-1-1) and M04M26 (mat1-2-1). The small red, green, and yellow dots represent fluorescent-labeled nuclei in separated mycelia. Yellow nucleus was detected from the heterokaryon, which was formed in paired cultured experiments of 24e and 26m strains. After the vegetative fusion, cytoplasm and organelles of two sexually compatible strains were integrated. eGFP or mCherry were expressed in cytoplasm and directed by nuclear localization signal into the same nucleus, resulting in the overlapping of red and green signals and the generation of yellow fluorescence signal in the merged image. The above schematic was generated by figdraw (www.figdraw.com).

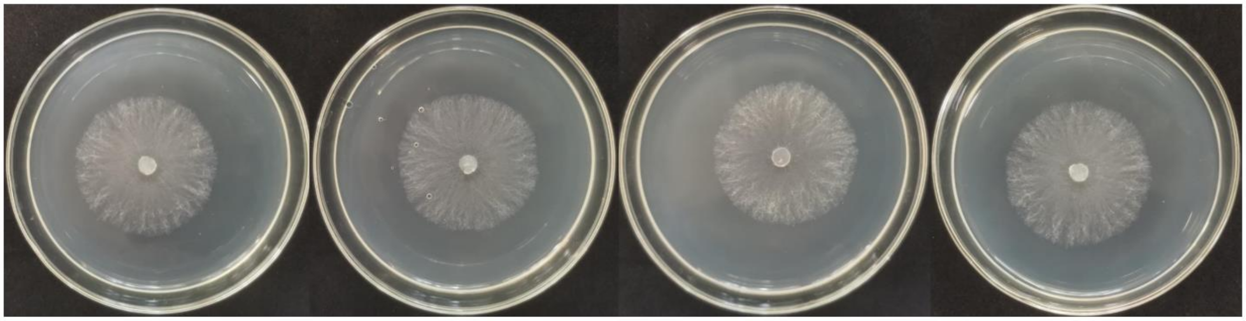

Supplementary Figure 2. Germination of *M. importuna* hyphae after 4-month storage at -80 ℃ in the 50% sterile glycerol-water solution (1:1, v/v).
